# A Census of Nuclear Cyanobacterial Recruits in the Plant Kingdom

**DOI:** 10.1371/journal.pone.0120527

**Published:** 2015-03-20

**Authors:** Szabolcs Makai, Xiao Li, Javeed Hussain, Cuiju Cui, Yuesheng Wang, Mingjie Chen, Zhaowan Yang, Chuang Ma, An-Yuan Guo, Yanhong Zhou, Junli Chang, Guangxiao Yang, Guangyuan He

**Affiliations:** 1 The Genetic Engineering International Cooperation Base of Chinese Ministry of Science and Technology, The Key Laboratory of Molecular Biophysics of Chinese Ministry of Education, College of Life Science and Technology, Huazhong University of Science & Technology, Wuhan, 430074, the People’s Republic of China; 2 Hubei Bioinformatics and Molecular Imaging Key Laboratory, College of Life Science and Technology, Huazhong University of Science & Technology, Wuhan, 430074, the People’s Republic of China; 3 Bioinformatics Laboratory, Applied Genomics Department, Agricultural Institute, Centre for Agricultural Research, Hungarian Academy of Sciences, Martonvásár, 2462, Hungary; Chinese Academy of Sciences, CHINA

## Abstract

The plastids and mitochondria of the eukaryotic cell are of endosymbiotic origin. These events occurred ~2 billion years ago and produced significant changes in the genomes of the host and the endosymbiont. Previous studies demonstrated that the invasion of land affected plastids and mitochondria differently and that the paths of mitochondrial integration differed between animals and plants. Other studies examined the reasons why a set of proteins remained encoded in the organelles and were not transferred to the nuclear genome. However, our understanding of the functional relations of the transferred genes is insufficient. In this paper, we report a high-throughput phylogenetic analysis to identify genes of cyanobacterial origin for plants of different levels of complexity: *Arabidopsis thaliana*, *Chlamydomonas reinhardtii*, *Physcomitrella patens*, *Populus trichocarpa*, *Selaginella moellendorffii*, *Sorghum bicolor*, *Oryza sativa*, and *Ostreococcus tauri*. Thus, a census of cyanobacterial gene recruits and a study of their function are presented to better understand the functional aspects of plastid symbiogenesis. From algae to angiosperms, the GO terms demonstrated a gradual expansion over functionally related genes in the nuclear genome, beginning with genes related to thylakoids and photosynthesis, followed by genes involved in metabolism, and finally with regulation-related genes, primarily in angiosperms. The results demonstrate that DNA is supplied to the nuclear genome on a permanent basis with no regard to function, and only what is needed is kept, which thereby expands on the GO space along the related genes.

## Introduction

Plastids and mitochondria are plant organelles originally derived from endosymbiotic bacteria [1]. Approximately 2 billion and 1.5 billion years of evolution of mitochondria and plastids, respectively, led to a close metabolic relationship between host and endosymbiont, and the translocation of most of the endosymbionts’ genetic material into the nuclear genome of the host. The genetic merger of two species is called symbiogenesis, as opposed to symbiosis [2, 3]. Endosymbiotic gene transfer (EGT) is an integral component of symbiogenesis and occurs in three stages. First, organelle DNA is integrated into the nuclear genome. Second, this DNA gains functionality by either retaining its original function or integrating into host-associated pathways [4–6]. At this stage, an adequate mechanism for translocation of the protein product is required as a prerequisite [7–12]. At the third stage, the original gene is lost or becomes a pseudogene [13]. A well-defined mechanistic model of this process was proposed for mitochondria, and the model shows that the process only stops when all coding DNA is transferred, which can ultimately lead to complete genome loss [14]. Indeed, such cases were reported in mitochondria [15]. Additionally, a considerable decrease of the genome size is observed for both organelles. The modern-day counterpart of the ancient α-proteobacterium, *Mesorhizobium loti*, harbors a genome of 7 Mb yet encodes more than 6,700 proteins, whereas the average number of protein coding genes of all sequenced mitochondrial genomes (chondriome) is only 3–67 [4, 16]. Similarly, the closest relative to modern-day plastids, *Nostoc PCC 7120*, has a genome of ~6.4 Mb which encodes ~5,400 proteins, whereas all sequenced plastomes encode only an average of 42–251 proteins [4, 17].

Many studies investigated why a subset of genes still remained in all plastids and mitochondria. Some hypotheses assume that the nucleus cannot take transcriptional control of these genes for several reasons. The hydrophobicity hypothesis does not adequately explain the retention of all organelle-encoded proteins because not all organelle encoded-proteins are hydrophobic [18]. The successful import of several hydrophobic nuclear-encoded chloroplast proteins, such as the light-harvesting chlorophyll *a/b*-binding proteins, conflicts with this hypothesis [19]. Protein import mechanisms primarily rely on specific molecular chaperones [20]. The presence of a vesicular transport system in chloroplasts conflicts with this hypothesis [21]. The CORR theory (CO-location for Redox Regulation) proposes a direct association between coding location and regulation; however, the expression of many nuclear-encoded organelle proteins is under redox control and yet they are not organelle-encoded [22, 23]. Furthermore, it was reported that mature leaves are fully functional with a chloroplast that contains only a fraction of their plastome [24] and that mRNA for the D1 protein (most prone to damage) is stable in the mature chloroplast [25].

The ‘limited transfer window’ hypothesis describes the reduced probability of DNA transfer in organisms with only a single organelle per cell [19, 26]. The hypothesis explains the low number of NUPTs (nuclear plastid DNA regions) in the nuclear genomes of *Chlamydomonas* and *Plasmodium* [27]. Nevertheless, this hypothesis does not eliminate the possibility of DNA transfer in such plants; rather, it describes an “inability to get them out” [19].

The non-protein coding genes are less affected by EGT, as there is no direct evidence of functional organelle-to-nuclear transfer of RNA genes. However, other evidence shows that the nuclear genes can replace organelle RNA genes. The mitochondrial genome of *Plasmodium* has lost all of its tRNA genes, and all necessary tRNAs are assumed to be imported from the cytosol [28]. In angiosperms and green algae, the genes for the RNA components of SRP and RNase P are absent, with the catalytic function transferred entirely to the protein component [29, 30].

The EGT continues, and according to the mechanistic models described above, it may end by transferring all organelle genes to the nucleus [31]. However, the EGT may appear as a slow or frozen process due to the enormous proliferation of angiosperms. On the other hand, however, also it causes all models that explain a requirement for a subset of proteins to remain in the organelles circumstantial [32].

In this comparative study, we analyzed the current situation of EGT and traced back to the ancient events of EGT. We looked both at their roles in biological processes and their cellular localization in the hope to better understand the functional aspects of symbiogenesis. A census of putative cyanobacterial recruits of plants is presented.

## Materials and Methods

### Data sources

The organelle and nuclear genomes were retrieved from EBI (http://www.ebi.ac.uk/), BIOL (http://merolae.biol.s.u-tokyo.ac.jp/), JGI (http://genome.jgi-psf.org/), NCBI (ftp://ftp.ncbi.nih.gov/genomes/), Phytozome (http://www.phytozome.net/), PlantDB (http://www.plantgdb.org/), JCVI (http://castorbean.jcvi.org/), and BGI (http://rice.genomics.org.cn/rice/index2.jsp/).

### Measure of DNA and gene transfer

The nuclear genome sequences of each plant species were aligned against their own plastomes and chondriomes by BLAST and NUCMER using a word size of 50 and a minimal length of exact matches of 50 bps, respectively [33, 34]. Both methods gave similar results. The hits were filtered for > 80% identity. The script to measure and chart coverage was written by one of the authors.

### Genome alignments

Genomes were aligned by the MAUVE Multiple Genome Aligner using progressive alignment and default settings [35].

### Phylogenetic analysis

The proteins encoded by each nuclear genome were assembled into a concatenated data set to perform BlastP, along with the cyanobacterial proteome and all 1,151 reference proteomes [36]. The BLAST hits were filtered for hits with E-values ≤ 10^−10^ and ≥ 25% amino acid identities. The extracted sequences from all selected proteins were aligned in MUSCLE (multiple sequence comparison by log-expectation) [37]. To give more significance to the probability distributions in the multiple sequence alignments, a maximum likelihood (ML) method was used, and more than 1,000 computationally tractable phylogenetic trees were generated in a JTT-F matrix using Tree-Puzzle 5.2 [38]. The nuclear and plastid genes of the selected species that had originated from cyanobacteria were identified from these output files (ML phylogenetic trees). BlastP was performed between the nuclear and plastid-encoded proteins of the selected species to identify gene transfers.

### Expression analysis

The aim of the analysis was to qualify, not quantify, expression; therefore, a simple method was used. The EST sequences were collected from NCBI, and the identified cyanobacterial recruits were queried with BLASTn (task: megablast) against them. If one or more hits were obtained, the gene was considered expressed.

### GO abundance study

Plant GOSlim terms were used to render a Voronoi tree map [39] using a software developed by one of the authors. The GO annotation files were obtained from public databases. The GO abundance analysis of GOSlim and complete GO terms were run on the AgriGO website (http://bioinfo.cau.edu.cn/agriGO/analysis.php). The coloring of the tree map is according to the multiple test adjusted *p*-values of each GO term.

## Results

### Cyanobacterial recruits

A complex, high-throughput phylogenetic analysis was conducted to identify possible cyanobacterial recruits in the nuclear genomes of plants. This study included over 1,000 reference proteomes in addition to the cyanobacterial proteomes to exclude cases of gene transfers from non-cyanobacteria. To build a representative census of putative cyanobacterial recruits of plants, four angiosperms (*Arabidopsis thaliana*, *Populus trichocarpa*, *Sorghum bicolor* and *Oryza*. *sativa)*, two algae (*Ostreococcus tauri* and *Chlamydomonas reinhardtii*), a bryophyte (*Physcomitrella patens*) and a lycophyte (*Selaginella moellendorffii*) were selected. The results are summarized in Tables 1 and 2. The numbers of nuclear-encoded proteins with putative cyanobacterial origin were, in all cases, greater than the numbers of nuclear encoded proteins homologous to plastid encoded proteins. This indicates the final stage of EGT, where the original genes are lost from the plastome. *P*. *trichocarpa* had the highest number of cyanobacterial gene recruits (835), followed by *P*. *patens* (823). Arabidopsis had 585 nuclear encoded proteins of putative cyanobacterial origin, and 53 homologous to 27 plastid-encoded proteins, whereas only one plastid coded protein was homologous to putatively cyanobacterial protein encoded in the nucleus (three copies were in the nuclear genome). By contrast, rice contained 482 cyanobacterial recruits in its nuclear genome, with 218 nuclear encoded proteins homologous to 55 plastid encoded ones, of which 29 plastid encoded proteins homologous to 68 nuclear recruits from cyanobacteria.

**Table 1:** The results of the census of proteins of cyanobacterial origin and nuclear-to-plastid gene homology.

Species	Nuclear genes putatively originated from cyanobacteria	Nuclear genes homologous with the plastid genes	Nuclear genes with putative cyanobacterial origin that are homologous with a plastid gene
*Arabidopsis thaliana*	585	53	3
*Oryza sativa*	482	218	68
*Sorghum bicolor*	538	68	15
*Populus trichocarpa*	835	136	43
*Physcomitrella patens*	823	96	19
*Selaginella moellendorffii*	350	110	4
*Chlamydomonas reinhardtii*	353	32	2
*Ostreococcus tauri*	233	58	3

**Table 2:** The plastid-to-nuclear gene homology.

Species	Plastid-encoded proteins	Plastid-encoded proteins homologous to at least one nuclear-encoded protein	Plastid-encoded proteins homologous to at least one nuclear-encoded protein of cyanobacterial origin
*Arabidopsis thaliana*	85	27	1
*Oryza sativa*	64	55	29
*Sorghum bicolor*	84	29	13
*Populus trichocarpa*	98	45	20
*Selaginella moellendorffii*	85	28	12
*Physcomitrella patens*	70	57	3
*Chlamydomonas reinhardtii*	69	15	1
*Ostreococcus tauri*	60	28	3

The *in vivo* expression of the identified cyanobacterial recruits was studied *in silico*. It was found that 98% of cyanobacterial recruits of *A*. *thaliana* were expressed, and slightly less than 82% were expressed in rice. In *S*. *bicolor*, 83% were expressed, and 64% were expressed in poplar. In *P*. *patens*, *S*. *moellendorffii*, *a*nd *C*. *reinhardtii*, 88%, 84%, and 79% of cyanobacterial recruits were expressed, respectively.

### Functional analysis of transferred genes

**Fig 1:**
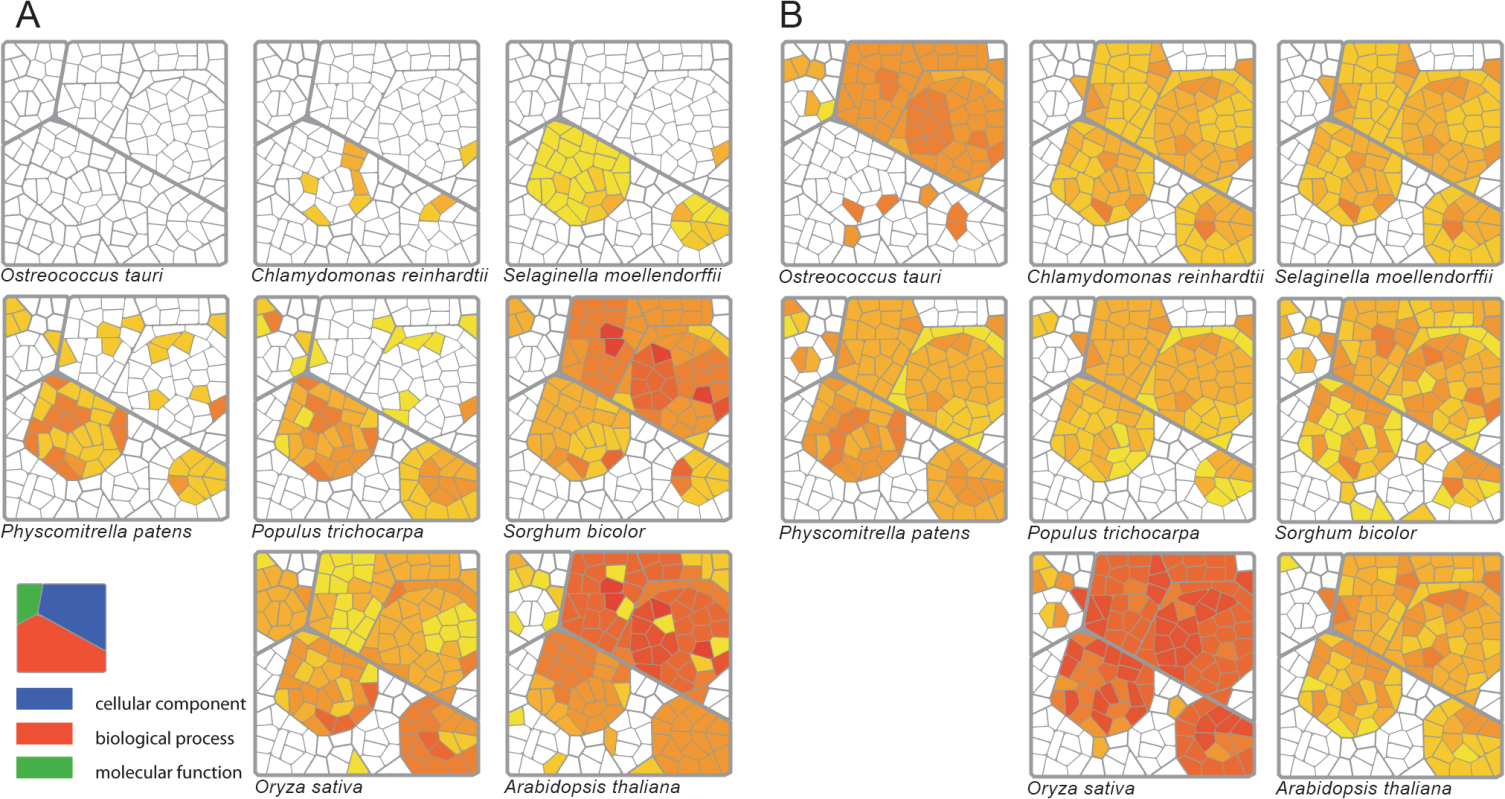
Voronoi treemap representation of the GO term enrichment study. (A) GO fingerprints of proteins of putative cyanobacterial origin of the eight species. A gradual invasion of the GO space by cyanobacterial recruits demonstrates the non-random nature of the GO terms distribution. This suggests a selection-driven gene transfer. (B) GO fingerprints of nuclear proteins with plastid homologues of the same eight species. No particular space is occupied by significant terms on any of the maps, which indicates that the nucleus acts as a DNA sink in all species, attracting genes in a wide range of GO terms.

For further analysis of the simple numbers of cyanobacterial recruits, an analysis of GO term abundance [21] was conducted to investigate the functional aspects of these genes (Fig. 1). The Plant GO Slim terms were mapped onto a Voronoi tree map [39] and were colored according to the significance values (Fig. 1A). The genes related to thylakoids (GO:0009579) and photosynthesis (GO:0015979) appeared in all species except in *O*. *tauri*, where no significant (p < 0.05) GO term was found among the cyanobacterial recruits. The genes related to nitrogen and lipid metabolism (GO:0006629 and GO:0006807, respectively) were also among the first to be transferred. Next, the genes with other metabolic- (GO:0006091, GO:0008152, and GO:0006519) and translation-related (GO:0006412) terms were transferred, as these terms appeared first on the GO maps of *S*. *moellendorffii* and *P*. *patens*. Additionally, genes related to ribosome (GO:0005840) and catalytic activity (including NADP binding, cofactor binding and lyase activity) appeared on the GO map of *P*. *patens*. Finally, as found for *A*. *thaliana* and *O*. *sativa*, response to abiotic stress (GO:0009628) and post-embryonic development (GO:0009791) related genes appeared on the GO map, and these genes demonstrated a close integration between the pathways of host and endosymbiont.

Additionally, the GO terms of cellular compartments demonstrated that the protein products of cyanobacterial recruits of *C*. *reinhardtii*, *S*. *moellendorffii*, and *P*. *patens* were localized primarily in the thylakoid and plastid, whereas in the cases of *S*. *bicolor*, *O*. *sativa*, and *A*. *thaliana*, the novel proteins were integrated into mitochondria and cytoplasm as well (Fig. 1A). These maps demonstrated a gradual expansion along a network of related terms both in the biological process (BP) and the cellular compartment (CC) groups of GO terms. This expansion stemmed from photosynthesis (GO:0015979) and thylakoid (GO:0009579) genes, respectively.

By contrast, the nuclear-encoded genes homologous to plastid-encoded genes presented less diverse GO term abundance profiles (Fig. 1B). The nuclear genomes had plastid homologue genes across a wide range of GO space, regardless of the species. Indeed, all eight species had similar profiles for nuclear-to-plastome homologue genes. Interestingly, the 58 nuclear-to-plastid homologue genes of *O*. *tauri* presented a GO abundance profile similar to that of *O*. *sativa*, whereas its 233 cyanobacterial recruits produced no significant GO term. The full list of the significant GO terms for each species is provided in S1 and S2 Tables.

The fully functional recruits represented genes at the third stage of EGT when the original gene was lost, whereas the plastid-to-nuclear homologues represented the first stage when DNA was transferred to the nucleus but was not functional. The former demonstrated systematic expansion of terms on the GO space, whereas the latter presented similar, nonsystematic distributions of significant terms on the GO space for the eight species. To further characterize the first and second stages of EGT, a comparative analysis of nuclear and organelle genomes was conducted.

### Characterization of genomes

We characterized the nuclear, plastid, and mitochondrial genomes by calculating the average and the variance of genome sizes, coding regions, and the numbers of protein-coding and functional genes of each phylogenetic group. These simple similarity based analyses indicated EGT in the first and/or second stage when DNA was integrated into the nuclear genome (where it might not be functional), whereas the original remained in the organelle. The chondriome was included to investigate earlier reports of different evolutionary paths for the plastome and the chondriome.

**Fig 2:**
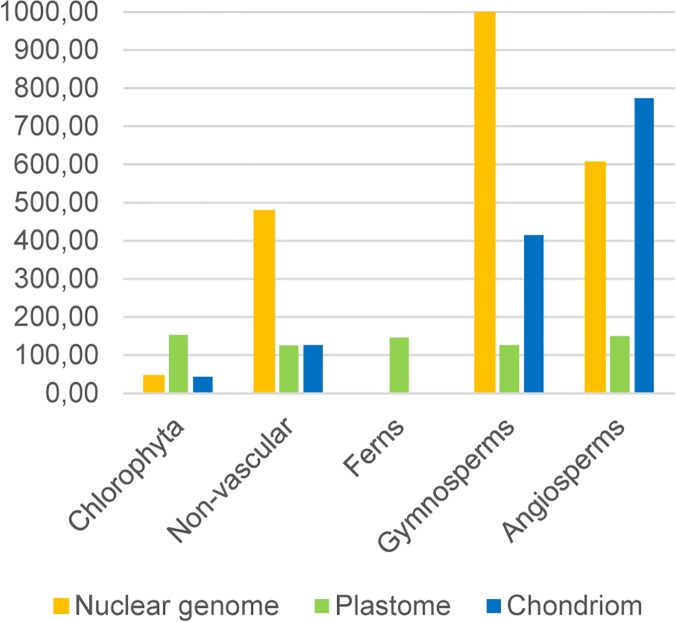
The average size in Mbps of the nuclear (orange), plastid (green) and mitochondrial (blue) genomes in phylogenetic groups. The plastome size remains similar in all the groups, whereas the chondriome seems to be inflating as plants are becoming more complex. The average genome size of gymnosperms is out of the scale due to the extremely large genome of Picea glauca, and the low number of species sequenced in the group.

The average size of the nuclear genome increased from algae (46 Mb) to angiosperms (605 Mb; Fig. 2). Compared with that of any other plant, the genome of algae was shorter. Additionally, protein-coding genes in the nucleus increased from few thousand in algae to more than 30,000 in angiosperms.

For all plants, the average size of the plastomes was similar (Fig. 2). However, between the groups, the variance of the plastid genome sizes was diverse (Table 3). Algae had the highest variance in plastome length, and the variance of plastome size decreased sharply from algae to land plants. The variance in number of proteins was high in algae and was low in angiosperms (Table 3); the exception was conifers, which revealed an unexpectedly large variance in the numbers of plastid-encoded proteins. The variance in the coding regions was almost two-fold higher for algae than for angiosperms. For all plants, the variance was similar for the number of RNAs.

**Table 3:** Relative standard deviations of plastome measures.

	Length (Mbp%)	Protein	RNA	Coding region
Chlorophyta	90.32	27.35	6.51	13.68
Nonvascular	14.51	8.39	0.70	5.91
Ferns	8.19	13.42	3.28	4.02
Gymnosperms	12.24	32.99	5.00	9.13
Angiosperms	16.96	10.32	5.57	7.22

In contrast to plastids, the size of the plant chondriome (Fig. 2) increased from algae to angiosperms, with lower variance in algae than in angiosperms (Table 4). For angiosperms, the variance was higher in the number of protein-coding genes than for nonvascular land plants and algae. The coding region of the chondriome decreased from 52% in algae to 15% in angiosperms, but the variance remained similar. However, the number of proteins coded by the chondriome increased with an increasing variance. The number of structural RNAs of the mitochondria remained similar in all species. The amount of noncoding DNA increased in all three cellular organelles (nucleus, plastid, and mitochondria), but the size of coding DNA increased concurrently only in the nucleus (data not shown).

**Table 4:** Relative standard variations of mitochondrial genome measures.

	Length (Mbp%)	Protein	RNA	Coding region
Chlorophyta	19.25	16.05	7.59	11.72
Nonvascular	34.22	11.44	2.31	9.26
Angiosperms	1676.51	43.20	6.13	13.36

### Similarities between nuclear and organelle genomes

The similarity between organelle and nuclear genomes of a species was determined where data were available. The lowest degree of similarity was observed in *C*. *reinhardtii* and the moss *P*. *paten*s, with values of 0.28% and 8.10%, respectively for the plastome, and 8.9% and 36.9%, respectively for the chondriome. The plastomes of angiosperms exhibited a high degree of similarity (60–100%) with their respective nuclear genomes, with the exception of *A*. *thaliana* (15.3%). Of the fully assembled genomes, the monocots had the highest transfer rate (> 90%). *Zea mays* had 99.4% of its plastome transferred into the nuclear genome, which was distributed across many chromosomes. In *S*. *moellendorffii*, 100% transfer was detected on a single scaffold (no. 175). This scaffold was reported to contain contamination of the organelle genome [40]; therefore, we excluded it from analysis, which reduced the calculated exchange rate to 15.7%.

Mitochondrial genomes presented a relatively low transfer rate. No complete chondriome was transferred, and the rates varied from 35.5% in *S*. *bicolor* to 94.4% in *Z*. *mays*. The numbers of NUMTs (nuclear mitochondrial DNA regions) and NUPTs were calculated for selected plant species (Tables 5 and 6). The NUPTs longer than 1,000 bps were found in great numbers among angiosperms, including 284 in *Z*. *mays*, 113 in *O*. *sativa* L. ssp. indica, and 111 in *Brachypodium distachyon*. More than 50 NUPTs longer than 1,000 bps were found in *Medicago truncatula*, *Vitis vinifera* and *Carica papaya*. In most species, most NUPTs were 100–200 bps long. Algae contained very few NUPTs. The longest NUPTs of *C*. *reinhardtii* and *Micromonas pusilla CCMP1545* had 1,060 and 2,124 bps, respectively. The analysis of NUMTs yielded similar results, as shown in Table 4.

**Table 5:** The number of NUPTs in the species studied.

			Size						
Species	Exchange	50–99		-199	-299	-399	-499	-999	≥ 1000
*Micromonas pusilla CCMP1545*	0.00%		1	3	2	0	0	0	1
*Chlamydomonas reinhardtii*	0.27%		9	0	0	0	0	0	0
*Ostreococcus tauri*	1.40%		0	1	0	1	0	0	0
*Physcomitrella patens*	7.70%		5	3	3	1	2	8	1
*Arabidopsis thaliana*	15.30%		55	26	12	3	5	12	3
*Selaginella moellendorffii*	15.60%		680	22	1	1	0	1	14
*Lotus japonicus*	60.16%		223	312	87	26	16	16	21
*Manihot esculenta*	61.33%		178	243	98	37	19	50	15
*Cucumis sativus*	63.35%		131	237	135	54	26	70	10
*Populus trichocarpa*	68.55%		112	381	166	65	41	40	22
*Prunus persica*	77.11%		383	358	108	62	30	38	41
*Sorghum bicolor*	80.31%		162	317	199	103	46	49	34
*Vitis vinifera*	84.03%		110	186	155	108	73	92	70
*Carica papaya*	93.45%		106	353	299	220	137	156	86
*Oryza sativa L*. ssp. Indica	94.37%		287	424	195	124	55	107	113
*Medicago truncatula*	95.69%		108	209	122	68	57	74	78
*Glycine max*	97.04%		438	938	673	355	153	131	48
*Brachypodium distachyon*	99.05%		208	403	325	207	83	72	111
*Zea mays*	99.51%		337	645	508	290	183	220	278

**Table 6:** The number of NUMTs in the species studied.

		Size						
Species	Exchange	50–99	-199	-299	-399	-499	-999	≥ 1000
*Ostreococcus tauri*	1%	0	0	0	0	0	1	0
*Chlamydomonas reinhardtii*	9%	1	4	1	2	0	0	0
*Sorghum bicolor*	35%	208	344	143	79	27	46	29
*Physcomitrella patens*	37%	20	33	15	7	3	11	15
*Carica papaya*	62%	138	274	175	141	77	117	68
*Vitis vinifera*	63%	183	336	183	89	56	113	184
*Oryza sativa L*. ssp. Indica	71%	1069	985	343	192	67	97	103
*Arabidopsis thaliana*	74%	127	61	20	10	4	8	14
*Cucumis sativus*	93%	431	269	25	4	0	8	18
*Zea mays*	94%	519	746	432	227	119	223	283

The copy number (CN) of putative transferred regions was calculated for angiosperms and *S*. *moellendorffii* as a potential further indicator of EGT. The results are shown in S1 and S2 Figs. The CN indicates how many times a given section of an organelle genome might have integrated in the nuclear genome. To investigate any regional preference of the transfer, the organelle genomes were aligned in locally collinear blocks (LCB). The results demonstrated that no LCBs had a preference for DNA transfer. However, the LCBs covered almost the complete length of most plastomes, whereas on the chondriomes, they covered only a small portion. This might further indicate that the inflation of the chondriome might be due to noncoding DNA material that diverges across species.

## Discussion

The increasing number of genome projects provides an opportunity to compare organelle genomes with their nuclear counterparts and to deepen our understanding of symbiogenesis. In the first part of our study, eight species were selected that represent different levels of complexity, and a census of their putative cyanobacterial recruits were assembled and functionally assessed. In the second part of the study, we conducted a comparative analysis of organelle genomes to gain insight into the level of similarity between organelle and nuclear genomes of plants.

The phylogenetic analysis demonstrated that *Arabidopsis* had a large number of genes of cyanobacterial origin that were not present in the plastome. A previous study concluded that 18% of the *A*. *thaliana* proteome was of cyanobacterial origin, a value higher than that reported in the present study [41]. The difference might be due to either the different genome release used in this study or to the different scales of the two phylogenetic studies. More than 1,000 reference proteomes were evaluated in the present study, in addition to the cyanobacteria proteome, whereas Martin et al. studied 17 reference proteomes and 15 chloroplasts. Another paper reported that approximately 14% of the nuclear-encoded proteins in *Arabidopsis*, rice, *C*. *reinhardtii*, and *Cyanidioschyzon* were of cyanobacterial origin [42]. Our analysis demonstrated that the rate of loss of the original gene from the organelle varied even among related species. Cycles of genome duplication and subsequent gene loss in plants offer one possible interpretation of these differences. *Arabidopsis* might have undergone more extensive “genome-cleaning” than poplar [43].

The fact that not all homologue genes in the nucleus are cyanobacterial recruits, suggests a non-cyanobacterial, exogenous origin. For example, in the green alga *O*. *tauri*, the genes on chromosome 19 do not share a significant phylogenetic relationship with other green algae, and many are weakly related to bacterial homologues. It was assumed that the entire chromosome might be derived from some exogenous source [44].

The story of EGT began 1.5 billion years ago and has not ended yet [31]. Closely after the second endosymbiotic event, plants started the conquest of land. The variance analysis of size, coding area, number of protein-coding genes, and number of structural genes produced very different results for the two organelles (high variance for the chondriome and low variance for the plastome), which indicates that plastids and mitochondria changed in opposite ways in plants. One reason for the high variance in plastome sizes within algae relative to angiosperms might be the seven distinct episodes of symbiogenesis as described by Keeling [45]. Additionally, algae inhabit a variety of unique ecosystems and might contain primary, secondary, tertiary, or serial secondary plastids which might contribute to the high variance in algal plastome size. Nevertheless, the variance profile of metazoan chondriomes was similar to that of the plastome in plants (low variance for both); therefore, perhaps, plastids are as important for plants as mitochondria are for metazoans, which further suggests that mitochondria of plants are under less selective pressure.

If the conquest of land was driven by competition for light and nitrogen, a tight integration of cyanobacterial pathways in the pathways of the host should come as no surprise. In the progression from algae to more complex plants, the relationship between the host and the symbiont became closer as was demonstrated by the functional analysis. Because “the genes related to photosynthesis were among the first to be transferred”, at the beginning of the symbiogenesis of the plastid, a translocation system must have been in operation. Then, when more complicated plants appeared, the gene pool of the plastid was a natural genetic resource for invention of novel pathways via EGT. But how exactly is EGT driven?

The cyanobacterial recruits in the nuclear genomes of algae were restricted to plastid-related functions, whereas in *Arabidopsis*, these genes were integrated in a wide spectrum of host-associated functions [5, 41]. Computational modeling of gene transfer for within-species-symbioses demonstrated that metabolically linked genes were more likely to be transferred [46]. The statistics of our study proved these observations were correct.

The figures illustrating patterns of GO abundance demonstrated that the nuclear genome attracted genes over a wide range of GO space (Fig. 1B), which suggested a continuous inflow of genes to the nucleus, regardless of function, which acted as a “gene-magnet”. However, these figures also confirmed that a full EGT was a nonrandom process. The GO abundance patterns of putative cyanobacterial recruits demonstrated a systematic expansion on the GO space, starting with genes related to photosynthesis in algae and then to genes that affected almost all biological processes and cellular compartments in higher plants. Therefore, the continuous inflow of DNA from the organelle presents a permanent and random supply of genetic material to the nucleus, whereas a changing environment and/or increasing complexity define the demands of the nucleus: “keeping only the useful genes”. To borrow a term from a market economy, endosymbiotic gene transfer behaved similar to a *demand-driven (genetic) supply chain* [47].

*Information is power*. The genome competence in plants increases with the transfer of hereditary information to the nucleus. Nuclear-encoded genes benefit from sexual recombination that enables the plant to adapt more quickly and effectively to changing environments. Through nuclear transcription, genes come under the control of complex and integrated processes that render plant cells able to initiate a coordinated response to any environmental change, wherein all cellular organelles can be activated from one main regulation center. Whereas energy production remains better optimized in the endosymbiotic-derived compartments, DNA moves out of the redox-load of organelles and relocates into a recombination-supporting environment. The endosymbiotic genetic potentials are rearranged such that some proteins may adopt novel roles [48]. Additionally, the DNA transfer to the nucleus provides the nucleus with the ability to functionally substitute a gene in the organelles; for example, *rps*13 was replaced by *rps*19 in *Arabidopsis* [49]. Finally, a significantly higher proportion of the organelle proteome is required for DNA maintenance and expression in the organelle, which can be saved by transferring the entire proteome to the nucleus. Consequently, we argue that the real question may not be why a subset of genes remained in organelles but what it requires to eliminate these genes via translocation into the nucleus.

## Supporting Information

S1 Table GO abundance study results for nuclear encoded proteins with putative cyanobacterial origin. Abbreviations: OT- *Ostreococcus tauri*, ChR—*Chlamydomonas reinhardtii*, SM—*Selaginella moellendorffii*, PP—*Physcomitrella patens*, PT—*Populus trichocarpa*, SB—*Sorghum bicolor*, OS—*Oryza_sativa*, AT—*Arabidopsis Thaliana*.
(PDF)

S2 Table GO abundance study results for nuclear encoded proteins with plastid encoded homologue. Abbreviations: OT- *Ostreococcus tauri*, ChR—*Chlamydomonas reinhardtii*, SM—*Selaginella moellendorffii*, PP—*Physcomitrella patens*, PT—*Populus trichocarpa*, SB—*Sorghum bicolor*, OS—*Oryza_sativa*, AT—*Arabidopsis Thaliana*.
(PDF)

S1 Fig Plastid genomes of angiosperms and *S*. *moelendorfii* aligned by MAUVE. Different colors represent locally collinear blocks detected by MAUVE. Orange diagram represents coverage plotted by nucleotide positions.
(TIF)

S2 Fig Mitochondrial genomes of angiosperms aligned by MAUVE. Different colors show locally collinear blocks (LCB as detected by MAUVE), oranges shows coverage as plotted against nucleotide positions.
(TIF)
