# Supplementary material for: A Census of Nuclear Cyanobacterial Recruits in the Plant Kingdom
Source: PLoS One. 2015 Mar 20;10(3):e0120527. doi: 10.1371/journal.pone.0120527 (PMC4368824; doi:10.1371/journal.pone.0120527)
Supplement: S1 Table — Abbreviations: OT- Ostreococcus tauri, ChR—Chlamydomonas reinhardtii, SM—Selaginella moellendorffii, PP—Physcomitrella patens, PT—Populus trichocarpa, SB—Sorghum bicolor, OS—Oryza_sativa, AT—Arabidopsis Thaliana. (PDF) [file pone.0120527.s001.pdf]

Supplementary table 1. GO abundance study results for nuclear encoded proteins with putative cyanobacterial origin. Abbreviations: OT- *Ostreococcus tauri* , ChR - *Chlamydomonas reinhardtii* , SM - *Selaginella moellendorffii* , PP - *Physcomitrella patens* , PT - *Populus trichocarpa* , SB - *Sorghum bicolor* , OS - *Oryza_sativa* , AT - *Arabidopsis Thaliana* .

GO Term	Aspect	Description	OT	ChR	SM	PP	PT	SB	OS	AT
GO:0033013	P	tetrapyrrole metabolic process								
GO:0033014	P	tetrapyrrole biosynthetic process								
GO:0006779	P	porphyrin biosynthetic process								
GO:0006778	P	porphyrin metabolic process								
GO:0051188	P	cofactor biosynthetic process								
GO:0046148	P	pigment biosynthetic process								
GO:0042440	P	pigment metabolic process								
GO:0034641	P	cellular nitrogen compound metabolic process								
GO:0051186	P	cofactor metabolic process								
GO:0044271	P	cellular nitrogen compound biosynthetic process								
GO:0018130	P	heterocycle biosynthetic process								
GO:0015994	P	chlorophyll metabolic process								
GO:0015995	P	chlorophyll biosynthetic process								
GO:0015979	P	photosynthesis								
GO:0046483	P	heterocycle metabolic process								
GO:0016053	P	organic acid biosynthetic process								
GO:0046394	P	carboxylic acid biosynthetic process								
GO:0043436	P	oxoacid metabolic process								
GO:0019752	P	carboxylic acid metabolic process								
GO:0006082	P	organic acid metabolic process								
GO:0042180	P	cellular ketone metabolic process								
GO:0006118	P	electron transport								
GO:0008610	P	lipid biosynthetic process								
GO:0044283	P	small molecule biosynthetic process								
GO:0044255	P	cellular lipid metabolic process								
GO:0044281	P	small molecule metabolic process								
GO:0044249	P	cellular biosynthetic process								
GO:0009058	P	biosynthetic process								
GO:0006520	P	cellular amino acid metabolic process								
GO:0008652	P	cellular amino acid biosynthetic process								
GO:0044106	P	cellular amine metabolic process								
GO:0009309	P	amine biosynthetic process								
GO:0008299	P	isoprenoid biosynthetic process								
GO:0006519	P	cellular amino acid and derivative metabolic process								
GO:0006720	P	isoprenoid metabolic process								
GO:0009308	P	amine metabolic process								
GO:0006629	P	lipid metabolic process								
GO:0006633	P	fatty acid biosynthetic process								
GO:0006631	P	fatty acid metabolic process								
GO:0006807	P	nitrogen compound metabolic process								
GO:0006790	P	sulfur metabolic process								
GO:0032787	P	monocarboxylic acid metabolic process								
GO:0005996	P	monosaccharide metabolic process								
GO:0006006	P	glucose metabolic process								
GO:0008152	P	metabolic process								
GO:0019318	P	hexose metabolic process								
GO:0016491	F	oxidoreductase activity								
GO:0051536	F	iron-sulfur cluster binding								
GO:0051540	F	metal cluster binding								
GO:0016705	F	oxidoreductase activity, acting on paired donors, with incorporation or reduction of molecular oxygen								
GO:0016835	F	carbon-oxygen lyase activity								
GO:0016836	F	hydro-lyase activity								
GO:0018600	F	alpha-pinene dehydrogenase activity								
GO:0018599	F	2-hydroxyisobutyrate 3-monooxygenase activity								
GO:0018598	F	hydroxymethylsilanetriol oxidase activity								
GO:0018591	F	methyl tertiary butyl ether 3-monooxygenase activity								
GO:0018590	F	methylsilanetriol hydroxylase activity								
GO:0018593	F	4-chlorophenoxyacetate monooxygenase activity								
GO:0018592	F	4-nitrocatechol 4-monooxygenase activity								
GO:0018595	F	alpha-pinene monooxygenase activity								
GO:0018597	F	ammonia monooxygenase activity								
GO:0018596	F	dimethylsilanediol hydroxylase activity								
GO:0004659	F	prenyltransferase activity								
GO:0020037	F	heme binding								
GO:0018594	F	tert-butyl alcohol 2-monooxygenase activity								
GO:0046906	F	tetrapyrrole binding								
GO:0018588	F	tri-n-butyltin dioxygenase activity								
GO:0018589	F	di-n-butyltin dioxygenase activity								
GO:0018586	F	mono-butyltin dioxygenase activity								
GO:0016829	F	lyase activity								
GO:0005506	F	iron ion binding								
GO:0018585	F	fluorene oxygenase activity								
GO:0043823	F	spheroidene monooxygenase activity								
GO:0016830	F	carbon-carbon lyase activity								
GO:0018587	F	limonene 8-monooxygenase activity								
GO:0009055	F	electron carrier activity								
GO:0016831	F	carboxy-lyase activity								
GO:0017171	F	serine hydrolase activity								
GO:0008236	F	serine-type peptidase activity								
GO:0034357	C	photosynthetic membrane								

GO Term	Aspect	Description	OT	ChR	SM	PP	PT	SB	OS	AT
GO:0009521	C	photosystem								
GO:0009579	C	thylakoid								
GO:0006725	P	cellular aromatic compound metabolic process								
GO:0006576	P	cellular biogenic amine metabolic process								
GO:0034637	P	cellular carbohydrate biosynthetic process								
GO:0006575	P	cellular amino acid derivative metabolic process								
GO:0009072	P	aromatic amino acid family metabolic process								
GO:0033692	P	cellular polysaccharide biosynthetic process								
GO:0000271	P	polysaccharide biosynthetic process								
GO:0016051	P	carbohydrate biosynthetic process								
GO:0044237	P	cellular metabolic process								
GO:0044264	P	cellular polysaccharide metabolic process								
GO:0006412	P	translation								
GO:0006091	P	generation of precursor metabolites and energy								
GO:0009987	P	cellular process								
GO:0006066	P	alcohol metabolic process								
GO:0018601	F	4-nitrophenol 2-monooxygenase activity								
GO:0051213	F	dioxygenase activity								
GO:0044436	C	thylakoid part								
GO:0009654	C	oxygen evolving complex								
GO:0019898	C	extrinsic to membrane								
GO:0009523	C	photosystem II								
GO:0055114	P	oxidation reduction								
GO:0006096	P	glycolysis								
GO:0015985	P	energy coupled proton transport, down electrochemical gradient								
GO:0015986	P	ATP synthesis coupled proton transport								
GO:0009206	P	purine ribonucleoside triphosphate biosynthetic process								
GO:0009201	P	ribonucleoside triphosphate biosynthetic process								
GO:0009142	P	nucleoside triphosphate biosynthetic process								
GO:0009145	P	purine nucleoside triphosphate biosynthetic process								
GO:0019320	P	hexose catabolic process								
GO:0046365	P	monosaccharide catabolic process								
GO:0006007	P	glucose catabolic process								
GO:0044275	P	cellular carbohydrate catabolic process								
GO:0006119	P	oxidative phosphorylation								
GO:0009152	P	purine ribonucleotide biosynthetic process								
GO:0046164	P	alcohol catabolic process								
GO:0009260	P	ribonucleotide biosynthetic process								
GO:0044282	P	small molecule catabolic process								
GO:0006164	P	purine nucleotide biosynthetic process								
GO:0019684	P	photosynthesis, light reaction								
GO:0009165	P	nucleotide biosynthetic process								
GO:0009116	P	nucleoside metabolic process								
GO:0016052	P	carbohydrate catabolic process								
GO:0006754	P	ATP biosynthetic process								
GO:0009199	P	ribonucleoside triphosphate metabolic process								
GO:0009144	P	purine nucleoside triphosphate metabolic process								
GO:0009205	P	purine ribonucleoside triphosphate metabolic process								
GO:0009141	P	nucleoside triphosphate metabolic process								
GO:0044262	P	cellular carbohydrate metabolic process								
GO:0055086	P	nucleobase, nucleoside and nucleotide metabolic process								
GO:0009150	P	purine ribonucleotide metabolic process								
GO:0046034	P	ATP metabolic process								
GO:0009259	P	ribonucleotide metabolic process								
GO:0006163	P	purine nucleotide metabolic process								
GO:0004332	F	fructose-bisphosphate aldolase activity								
GO:0016832	F	aldehyde-lyase activity								
GO:0010277	F	chlorophyllide a oxygenase activity								
GO:0003824	F	catalytic activity								
GO:0051537	F	2 iron, 2 sulfur cluster binding								
GO:0016703	F	oxidoreductase activity, acting on single donors with incorporation of molecular oxy								
GO:0004497	F	monooxygenase activity								
GO:0005198	F	structural molecule activity								
GO:0016717	F	oxidoreductase activity, acting on paired donors, with oxidation of a pair of donors r								
GO:0016903	F	oxidoreductase activity, acting on the aldehyde or oxo group of donors								
GO:0003735	F	structural constituent of ribosome								
GO:0009538	C	photosystem I reaction center								
GO:0009522	C	photosystem I								
GO:0032991	C	macromolecular complex								
GO:0009536	C	plastid								
GO:0045259	C	proton-transporting ATP synthase complex								
GO:0009507	C	chloroplast								
GO:0043234	C	protein complex								
GO:0005840	C	ribosome								
GO:0044424	C	intracellular part								
GO:0030529	C	ribonucleoprotein complex								
GO:0006783	P	heme biosynthetic process								
GO:0042168	P	heme metabolic process								
GO:0030243	P	cellulose metabolic process								
GO:0030244	P	cellulose biosynthetic process								
GO:0009250	P	glucan biosynthetic process								
GO:0005975	P	carbohydrate metabolic process								
GO:0044238	P	primary metabolic process								
GO:0006073	P	cellular glucan metabolic process								
GO:0044042	P	glucan metabolic process								

GO Term	Aspect	Description	OT	ChR	SM	PP	PT	SB	OS	AT
GO:0043648	P	dicarboxylic acid metabolic process								
GO:0005976	P	polysaccharide metabolic process								
GO:0006281	P	DNA repair								
GO:0044265	P	cellular macromolecule catabolic process								
GO:0006974	P	response to DNA damage stimulus								
GO:0051716	P	cellular response to stimulus								
GO:0033554	P	cellular response to stress								
GO:0006259	P	DNA metabolic process								
GO:0044248	P	cellular catabolic process								
GO:0034660	P	ncRNA metabolic process								
GO:0009057	P	macromolecule catabolic process								
GO:0006418	P	tRNA aminoacylation for protein translation								
GO:0043038	P	amino acid activation								
GO:0043039	P	tRNA aminoacylation								
GO:0009117	P	nucleotide metabolic process								
GO:0006753	P	nucleoside phosphate metabolic process								
GO:0006399	P	tRNA metabolic process								
GO:0019538	P	protein metabolic process								
GO:0006457	P	protein folding								
GO:0045454	P	cell redox homeostasis								
GO:0004325	F	ferrochelatase activity								
GO:0016854	F	racemase and epimerase activity								
GO:0016760	F	cellulose synthase (UDP-forming) activity								
GO:0016759	F	cellulose synthase activity								
GO:0016853	F	isomerase activity								
GO:0046527	F	glucosyltransferase activity								
GO:0035251	F	UDP-glucosyltransferase activity								
GO:0016857	F	racemase and epimerase activity, acting on carbohydrates and derivatives								
GO:0004750	F	ribulose-phosphate 3-epimerase activity								
GO:0016701	F	oxidoreductase activity, acting on single donors with incorporation of molecular oxy								
GO:0008194	F	UDP-glycosyltransferase activity								
GO:0003913	F	DNA photolyase activity								
GO:0016740	F	transferase activity								
GO:0016887	F	ATPase activity								
GO:0048037	F	cofactor binding								
GO:0015299	F	solute:hydrogen antiporter activity								
GO:0015298	F	solute:cation antiporter activity								
GO:0015300	F	solute:solute antiporter activity								
GO:0016616	F	oxidoreductase activity, acting on the CH-OH group of donors, NAD or NADP as acc								
GO:0004222	F	metalloendopeptidase activity								
GO:0016817	F	hydrolase activity, acting on acid anhydrides								
GO:0016614	F	oxidoreductase activity, acting on CH-OH group of donors								
GO:0016779	F	nucleotidyltransferase activity								
GO:0005515	F	protein binding								
GO:0017111	F	nucleoside-triphosphatase activity								
GO:0050662	F	coenzyme binding								
GO:0004812	F	aminoacyl-tRNA ligase activity								
GO:0016875	F	ligase activity, forming carbon-oxygen bonds								
GO:0016876	F	ligase activity, forming aminoacyl-tRNA and related compounds								
GO:0016462	F	pyrophosphatase activity								
GO:0005509	F	calcium ion binding								
GO:0016818	F	hydrolase activity, acting on acid anhydrides, in phosphorus-containing anhydrides								
GO:0000166	F	nucleotide binding								
GO:0031072	F	heat shock protein binding								
GO:0070279	F	vitamin B6 binding								
GO:0030170	F	pyridoxal phosphate binding								
GO:0070011	F	peptidase activity, acting on L-amino acid peptides								
GO:0008237	F	metallopeptidase activity								
GO:0019867	C	outer membrane								
GO:0016020	C	membrane								
GO:0019685	P	photosynthesis, dark reaction								
GO:0019253	P	reductive pentose-phosphate cycle								
GO:0015977	P	carbon fixation								
GO:0009657	P	plastid organization								
GO:0009658	P	chloroplast organization								
GO:0016108	P	tetraterpenoid metabolic process								
GO:0016116	P	carotenoid metabolic process								
GO:0016109	P	tetraterpenoid biosynthetic process								
GO:0016117	P	carotenoid biosynthetic process								
GO:0009767	P	photosynthetic electron transport chain								
GO:0006098	P	pentose-phosphate shunt								
GO:0006740	P	NADPH regeneration								
GO:0016114	P	terpenoid biosynthetic process								
GO:0006739	P	NADP metabolic process								
GO:0006733	P	oxidoreduction coenzyme metabolic process								
GO:0022900	P	electron transport chain								
GO:0019362	P	pyridine nucleotide metabolic process								
GO:0042732	P	D-xylose metabolic process								
GO:0046417	P	chorismate metabolic process								
GO:0009073	P	aromatic amino acid family biosynthetic process								
GO:0009110	P	vitamin biosynthetic process								
GO:0006721	P	terpenoid metabolic process								
GO:0046496	P	nicotinamide nucleotide metabolic process								
GO:0006769	P	nicotinamide metabolic process								
GO:0046383	P	dTDP-rhamnose metabolic process								

GO Term	Aspect	Description	OT	ChR	SM	PP	PT	SB	OS	AT
GO:0019300	P	rhamnose biosynthetic process								
GO:0019305	P	dTDP-rhamnose biosynthetic process								
GO:0019299	P	rhamnose metabolic process								
GO:0009266	P	response to temperature stimulus								
GO:0000162	P	tryptophan biosynthetic process								
GO:0046219	P	indolalkylamine biosynthetic process								
GO:0009820	P	alkaloid metabolic process								
GO:0019740	P	nitrogen utilization								
GO:0006808	P	regulation of nitrogen utilization								
GO:0043603	P	cellular amide metabolic process								
GO:0045036	P	protein targeting to chloroplast								
GO:0045037	P	protein import into chloroplast stroma								
GO:0009409	P	response to cold								
GO:0010189	P	vitamin E biosynthetic process								
GO:0042360	P	vitamin E metabolic process								
GO:0019321	P	pentose metabolic process								
GO:0019252	P	starch biosynthetic process								
GO:0006568	P	tryptophan metabolic process								
GO:0006586	P	indolalkylamine metabolic process								
GO:0006766	P	vitamin metabolic process								
GO:0010020	P	chloroplast fission								
GO:0043572	P	plastid fission								
GO:0042362	P	fat-soluble vitamin biosynthetic process								
GO:0005978	P	glycogen biosynthetic process								
GO:0009226	P	nucleotide-sugar biosynthetic process								
GO:0042401	P	cellular biogenic amine biosynthetic process								
GO:0043155	P	negative regulation of photosynthesis, light reaction								
GO:0010205	P	photoinhibition								
GO:0042777	P	plasma membrane ATP synthesis coupled proton transport								
GO:0031163	P	metallo-sulfur cluster assembly								
GO:0016226	P	iron-sulfur cluster assembly								
GO:0042435	P	indole derivative biosynthetic process								
GO:0006775	P	fat-soluble vitamin metabolic process								
GO:0005977	P	glycogen metabolic process								
GO:0006732	P	coenzyme metabolic process								
GO:0005982	P	starch metabolic process								
GO:0006112	P	energy reserve metabolic process								
GO:0042364	P	water-soluble vitamin biosynthetic process								
GO:0042434	P	indole derivative metabolic process								
GO:0042430	P	indole and derivative metabolic process								
GO:0006510	P	ATP-dependent proteolysis								
GO:0042548	P	regulation of photosynthesis, light reaction								
GO:0009225	P	nucleotide-sugar metabolic process								
GO:0010109	P	regulation of photosynthesis								
GO:0019748	P	secondary metabolic process								
GO:0019319	P	hexose biosynthetic process								
GO:0046246	P	terpene biosynthetic process								
GO:0009644	P	response to high light intensity								
GO:0034285	P	response to disaccharide stimulus								
GO:0009744	P	response to sucrose stimulus								
GO:0006090	P	pyruvate metabolic process								
GO:0046364	P	monosaccharide biosynthetic process								
GO:0006767	P	water-soluble vitamin metabolic process								
GO:0015980	P	energy derivation by oxidation of organic compounds								
GO:0042214	P	terpene metabolic process								
GO:0046165	P	alcohol biosynthetic process								
GO:0043467	P	regulation of generation of precursor metabolites and energy								
GO:0008654	P	phospholipid biosynthetic process								
GO:0010027	P	thylakoid membrane organization								
GO:0009668	P	plastid membrane organization								
GO:0009628	P	response to abiotic stimulus								
GO:0009642	P	response to light intensity								
GO:0034220	P	ion transmembrane transport								
GO:0007186	P	G-protein coupled receptor protein signaling pathway								
GO:0009416	P	response to light stimulus								
GO:0008878	F	glucose-1-phosphate adenylyltransferase activity								
GO:0015231	F	5-formyltetrahydrofolate transporter activity								
GO:0048040	F	UDP-glucuronate decarboxylase activity								
GO:0008460	F	dTDP-glucose 4,6-dehydratase activity								
GO:0047100	F	glyceraldehyde-3-phosphate dehydrogenase (NADP+) (phosphorylating) activity								
GO:0004056	F	argininosuccinate lyase activity								
GO:0008886	F	glyceraldehyde-3-phosphate dehydrogenase (NADP+) activity								
GO:0003959	F	NADPH dehydrogenase activity								
GO:0042389	F	omega-3 fatty acid desaturase activity								
GO:0008517	F	folic acid transporter activity								
GO:0004176	F	ATP-dependent peptidase activity								
GO:0016744	F	transferase activity, transferring aldehyde or ketonic groups								
GO:0004618	F	phosphoglycerate kinase activity								
GO:0008487	F	prenyl-dependent CAAX protease activity								
GO:0004802	F	transketolase activity								
GO:0016842	F	amidine-lyase activity								
GO:0051183	F	vitamin transporter activity								
GO:0004337	F	geranyltranstransferase activity								
GO:0004834	F	tryptophan synthase activity								
GO:0070566	F	adenylyltransferase activity								

GO Term	Aspect	Description	OT	ChR	SM	PP	PT	SB	OS	AT
GO:0004564	F	beta-fructofuranosidase activity								
GO:0016765	F	transferase activity, transferring alkyl or aryl (other than methyl) groups								
GO:0051184	F	cofactor transporter activity								
GO:0004365	F	glyceraldehyde-3-phosphate dehydrogenase (phosphorylating) activity								
GO:0008943	F	glyceraldehyde-3-phosphate dehydrogenase activity								
GO:0016774	F	phosphotransferase activity, carboxyl group as acceptor								
GO:0016984	F	ribulose-bisphosphate carboxylase activity								
GO:0016730	F	oxidoreductase activity, acting on iron-sulfur proteins as donors								
GO:0008266	F	poly(U) RNA binding								
GO:0016620	F	oxidoreductase activity, acting on the aldehyde or oxo group of donors, NAD or NAD[illegible]								
GO:0004033	F	aldo-keto reductase activity								
GO:0000287	F	magnesium ion binding								
GO:0016651	F	oxidoreductase activity, acting on NADH or NADPH								
GO:0051287	F	NAD or NADH binding								
GO:0008187	F	poly-pyrimidine tract binding								
GO:0050661	F	NADP or NADPH binding								
GO:0016840	F	carbon-nitrogen lyase activity								
GO:0016627	F	oxidoreductase activity, acting on the CH-CH group of donors								
GO:0003727	F	single-stranded RNA binding								
GO:0019842	F	vitamin binding								
GO:0016860	F	intramolecular oxidoreductase activity								
GO:0016861	F	intramolecular oxidoreductase activity, interconverting aldoses and ketoses								
GO:0050660	F	FAD binding								
GO:0022890	F	inorganic cation transmembrane transporter activity								
GO:0042625	F	ATPase activity, coupled to transmembrane movement of ions								
GO:0044435	C	plastid part								
GO:0044434	C	chloroplast part								
GO:0009534	C	chloroplast thylakoid								
GO:0031976	C	plastid thylakoid								
GO:0042651	C	thylakoid membrane								
GO:0009535	C	chloroplast thylakoid membrane								
GO:0055035	C	plastid thylakoid membrane								
GO:0031984	C	organelle subcompartment								
GO:0044444	C	cytoplasmic part								
GO:0010287	C	plastoglobule								
GO:0031090	C	organelle membrane								
GO:0009532	C	plastid stroma								
GO:0005737	C	cytoplasm								
GO:0009526	C	plastid envelope								
GO:0009570	C	chloroplast stroma								
GO:0009941	C	chloroplast envelope								
GO:0042170	C	plastid membrane								
GO:0043231	C	intracellular membrane-bounded organelle								
GO:0043227	C	membrane-bounded organelle								
GO:0009528	C	plastid inner membrane								
GO:0009706	C	chloroplast inner membrane								
GO:0031969	C	chloroplast membrane								
GO:0030075	C	plasma membrane-derived thylakoid								
GO:0031978	C	plastid thylakoid lumen								
GO:0031977	C	thylakoid lumen								
GO:0009543	C	chloroplast thylakoid lumen								
GO:0043229	C	intracellular organelle								
GO:0043226	C	organelle								
GO:0044446	C	intracellular organelle part								
GO:0044422	C	organelle part								
GO:0031975	C	envelope								
GO:0005622	C	intracellular								
GO:0030096	C	plasma membrane-derived thylakoid photosystem II								
GO:0031967	C	organelle envelope								
GO:0030094	C	plasma membrane-derived photosystem I								
GO:0009501	C	amyloplast								
GO:0030095	C	chloroplast photosystem II								
GO:0009842	C	cyanelle								
GO:0044464	C	cell part								
GO:0005623	C	cell								
GO:0019866	C	organelle inner membrane								
GO:0006120	P	mitochondrial electron transport, NADH to ubiquinone								
GO:0022904	P	respiratory electron transport chain								
GO:0042773	P	ATP synthesis coupled electron transport								
GO:0042775	P	mitochondrial ATP synthesis coupled electron transport								
GO:0009082	P	branched chain family amino acid biosynthetic process								
GO:0006818	P	hydrogen transport								
GO:0015992	P	proton transport								
GO:0045333	P	cellular respiration								
GO:0009081	P	branched chain family amino acid metabolic process								
GO:0055085	P	transmembrane transport								
GO:0015672	P	monovalent inorganic cation transport								
GO:0009161	P	ribonucleoside monophosphate metabolic process								
GO:0009156	P	ribonucleoside monophosphate biosynthetic process								
GO:0043170	P	macromolecule metabolic process								
GO:0006811	P	ion transport								
GO:0009123	P	nucleoside monophosphate metabolic process								
GO:0009124	P	nucleoside monophosphate biosynthetic process								
GO:0006812	P	cation transport								
GO:0009084	P	glutamine family amino acid biosynthetic process								

GO Term	Aspect	Description	OT	ChR	SM	PP	PT	SB	OS	AT
GO:0016310	P	phosphorylation								
GO:0006644	P	phospholipid metabolic process								
GO:0009064	P	glutamine family amino acid metabolic process								
GO:0019637	P	organophosphate metabolic process								
GO:0009056	P	catabolic process								
GO:0006796	P	phosphate metabolic process								
GO:0006793	P	phosphorus metabolic process								
GO:0006396	P	RNA processing								
GO:0044260	P	cellular macromolecule metabolic process								
GO:0034470	P	ncRNA processing								
GO:0065008	P	regulation of biological quality								
GO:0008033	P	tRNA processing								
GO:0042592	P	homeostatic process								
GO:0044267	P	cellular protein metabolic process								
GO:0003954	F	NADH dehydrogenase activity								
GO:0016655	F	oxidoreductase activity, acting on NADH or NADPH, quinone or similar compound as								
GO:0050136	F	NADH dehydrogenase (quinone) activity								
GO:0008137	F	NADH dehydrogenase (ubiquinone) activity								
GO:0004749	F	ribose phosphate diphosphokinase activity								
GO:0008937	F	ferredoxin reductase activity								
GO:0016731	F	oxidoreductase activity, acting on iron-sulfur proteins as donors, NAD or NADP as a								
GO:0016778	F	diphosphotransferase activity								
GO:0016820	F	hydrolase activity, acting on acid anhydrides, catalyzing transmembrane movement								
GO:0004324	F	ferredoxin-NADP+ reductase activity								
GO:0046933	F	hydrogen ion transporting ATP synthase activity, rotational mechanism								
GO:0046961	F	proton-transporting ATPase activity, rotational mechanism								
GO:0003984	F	acetolactate synthase activity								
GO:0004765	F	shikimate kinase activity								
GO:0015078	F	hydrogen ion transmembrane transporter activity								
GO:0019829	F	cation-transporting ATPase activity								
GO:0016772	F	transferase activity, transferring phosphorus-containing groups								
GO:0016787	F	hydrolase activity								
GO:0042623	F	ATPase activity, coupled								
GO:0017076	F	purine nucleotide binding								
GO:0022857	F	transmembrane transporter activity								
GO:0015077	F	monovalent inorganic cation transmembrane transporter activity								
GO:0016628	F	oxidoreductase activity, acting on the CH-CH group of donors, NAD or NADP as acc								
GO:0043492	F	ATPase activity, coupled to movement of substances								
GO:0015405	F	P-P-bond-hydrolysis-driven transmembrane transporter activity								
GO:0015399	F	primary active transmembrane transporter activity								
GO:0042626	F	ATPase activity, coupled to transmembrane movement of substances								
GO:0030554	F	adenyl nucleotide binding								
GO:0001883	F	purine nucleoside binding								
GO:0001882	F	nucleoside binding								
GO:0009003	F	signal peptidase activity								
GO:0015075	F	ion transmembrane transporter activity								
GO:0022891	F	substrate-specific transmembrane transporter activity								
GO:0051539	F	4 iron, 4 sulfur cluster binding								
GO:0005488	F	binding								
GO:0022892	F	substrate-specific transporter activity								
GO:0032555	F	purine ribonucleotide binding								
GO:0032553	F	ribonucleotide binding								
GO:0008324	F	cation transmembrane transporter activity								
GO:0016874	F	ligase activity								
GO:0005524	F	ATP binding								
GO:0032559	F	adenyl ribonucleotide binding								
GO:0022804	F	active transmembrane transporter activity								
GO:0005215	F	transporter activity								
GO:0022836	F	gated channel activity								
GO:0004175	F	endopeptidase activity								
GO:0043176	F	amine binding								
GO:0016597	F	amino acid binding								
GO:0015662	F	ATPase activity, coupled to transmembrane movement of ions, phosphorylative me								
GO:0016667	F	oxidoreductase activity, acting on sulfur group of donors								
GO:0031406	F	carboxylic acid binding								
GO:0022838	F	substrate-specific channel activity								
GO:0022803	F	passive transmembrane transporter activity								
GO:0005216	F	ion channel activity								
GO:0015267	F	channel activity								
GO:0044425	C	membrane part								
GO:0016469	C	proton-transporting two-sector ATPase complex								
GO:0016021	C	integral to membrane								
GO:0031224	C	intrinsic to membrane								
GO:0043232	C	intracellular non-membrane-bounded organelle								
GO:0043228	C	non-membrane-bounded organelle								
GO:0070271	P	protein complex biogenesis								
GO:0006461	P	protein complex assembly								
GO:0043623	P	cellular protein complex assembly								
GO:0046493	P	lipid A metabolic process								
GO:0009245	P	lipid A biosynthetic process								
GO:0008653	P	lipopolysaccharide metabolic process								
GO:0009103	P	lipopolysaccharide biosynthetic process								
GO:0017004	P	cytochrome complex assembly								
GO:0043933	P	macromolecular complex subunit organization								
GO:0034621	P	cellular macromolecular complex subunit organization								

GO Term	Aspect	Description	OT	ChR	SM	PP	PT	SB	OS	AT
GO:0010190	P	cytochrome b6f complex assembly								
GO:0065003	P	macromolecular complex assembly								
GO:0005986	P	sucrose biosynthetic process								
GO:0005985	P	sucrose metabolic process								
GO:0034622	P	cellular macromolecular complex assembly								
GO:0009791	P	post-embryonic development								
GO:0009247	P	glycolipid biosynthetic process								
GO:0044085	P	cellular component biogenesis								
GO:0009098	P	leucine biosynthetic process								
GO:0022607	P	cellular component assembly								
GO:0006664	P	glycolipid metabolic process								
GO:0043085	P	positive regulation of catalytic activity								
GO:0044093	P	positive regulation of molecular function								
GO:0016043	P	cellular component organization								
GO:0046467	P	membrane lipid biosynthetic process								
GO:0046351	P	disaccharide biosynthetic process								
GO:0009312	P	oligosaccharide biosynthetic process								
GO:0006551	P	leucine metabolic process								
GO:0005984	P	disaccharide metabolic process								
GO:0006643	P	membrane lipid metabolic process								
GO:0009311	P	oligosaccharide metabolic process								
GO:0009793	P	embryonic development ending in seed dormancy								
GO:0009790	P	embryonic development								
GO:0045426	P	quinone cofactor biosynthetic process								
GO:0042375	P	quinone cofactor metabolic process								
GO:0006996	P	organelle organization								
GO:0009314	P	response to radiation								
GO:0009408	P	response to heat								
GO:0010154	P	fruit development								
GO:0048316	P	seed development								
GO:0048481	P	ovule development								
GO:0016138	P	glycoside biosynthetic process								
GO:0003006	P	reproductive developmental process								
GO:0048608	P	reproductive structure development								
GO:0010035	P	response to inorganic substance								
GO:0048440	P	carpel development								
GO:0006979	P	response to oxidative stress								
GO:0010038	P	response to metal ion								
GO:0046686	P	response to cadmium ion								
GO:0009108	P	coenzyme biosynthetic process								
GO:0019220	P	regulation of phosphate metabolic process								
GO:0051174	P	regulation of phosphorus metabolic process								
GO:0048467	P	gynoecium development								
GO:0016137	P	glycoside metabolic process								
GO:0050307	F	sucrose-phosphatase activity								
GO:0008759	F	UDP-3-O-[3-hydroxymyristoyl] N-acetylglucosamine deacetylase activity								
GO:0004311	F	farnesyltranstransferase activity								
GO:0016630	F	protochlorophyllide reductase activity								
GO:0003862	F	3-isopropylmalate dehydrogenase activity								
GO:0019213	F	deacetylase activity								
GO:0004605	F	phosphatidate cytidylyltransferase activity								
GO:0070567	F	cytidylyltransferase activity								
GO:0008415	F	acyltransferase activity								
GO:0016747	F	transferase activity, transferring acyl groups other than amino-acyl groups								
GO:0005319	F	lipid transporter activity								
GO:0016407	F	acetyltransferase activity								
GO:0016746	F	transferase activity, transferring acyl groups								
GO:0008080	F	N-acetyltransferase activity								
GO:0008233	F	peptidase activity								
GO:0016410	F	N-acyltransferase activity								
GO:0003746	F	translation elongation factor activity								
GO:0016811	F	hydrolase activity, acting on carbon-nitrogen (but not peptide) bonds, in linear ami								
GO:0008168	F	methyltransferase activity								
GO:0016741	F	transferase activity, transferring one-carbon groups								
GO:0008171	F	O-methyltransferase activity								
GO:0046915	F	transition metal ion transmembrane transporter activity								
GO:0008047	F	enzyme activator activity								
GO:0004553	F	hydrolase activity, hydrolyzing O-glycosyl compounds								
GO:0016810	F	hydrolase activity, acting on carbon-nitrogen (but not peptide) bonds								
GO:0010319	C	stromule								
GO:0010007	C	magnesium chelatase complex								
GO:0010598	C	NAD(P)H dehydrogenase complex (plastoquinone)								
GO:0000311	C	plastid large ribosomal subunit								
GO:0000315	C	organellar large ribosomal subunit								
GO:0070013	C	intracellular organelle lumen								
GO:0043233	C	organelle lumen								
GO:0031974	C	membrane-enclosed lumen								
GO:0009547	C	plastid ribosome								
GO:0009527	C	plastid outer membrane								
GO:0009707	C	chloroplast outer membrane								
GO:0000313	C	organellar ribosome								
GO:0031968	C	organelle outer membrane								
