# Supplementary material for: A Census of Nuclear Cyanobacterial Recruits in the Plant Kingdom
Source: PLoS One. 2015 Mar 20;10(3):e0120527. doi: 10.1371/journal.pone.0120527 (PMC4368824; doi:10.1371/journal.pone.0120527)
Supplement: S2 Table — Abbreviations: OT- Ostreococcus tauri, ChR—Chlamydomonas reinhardtii, SM—Selaginella moellendorffii, PP—Physcomitrella patens, PT—Populus trichocarpa, SB—Sorghum bicolor, OS—Oryza_sativa, AT—Arabidopsis Thaliana. (PDF) [file pone.0120527.s002.pdf]

Supplementary table 2. GO abundance study results for nuclear encoded proteins with plastid encoded homologue. Abbreviations: OT- *Ostreococcus tauri* , ChR - *Chlamydomonas reinhardtii* , SM - *Selaginella moellendorffii* , PP - *Physcomitrella patens* , PT - *Populus trichocarpa* , SB - *Sorghum_bicolor* , OS - *Oryza sativa* , AT - *Arabidopsis Thaliana* .

GO Term	Aspec	Description	OT	ChR	SM	PP	PT	SB	OS	AT
GO:0006091	P	generation of precursor metabolites and energy								
GO:0015979	P	photosynthesis								
GO:0022900	P	electron transport chain								
GO:0034220	P	ion transmembrane transport								
GO:0015992	P	proton transport								
GO:0006818	P	hydrogen transport								
GO:0015985	P	energy coupled proton transport, down electrochemical gradient								
GO:0015986	P	ATP synthesis coupled proton transport								
GO:0006119	P	oxidative phosphorylation								
GO:0006754	P	ATP biosynthetic process								
GO:0009142	P	nucleoside triphosphate biosynthetic process								
GO:0009145	P	purine nucleoside triphosphate biosynthetic process								
GO:0015672	P	monovalent inorganic cation transport								
GO:0009206	P	purine ribonucleoside triphosphate biosynthetic process								
GO:0009201	P	ribonucleoside triphosphate biosynthetic process								
GO:0009767	P	photosynthetic electron transport chain								
GO:0006810	P	transport								
GO:0051234	P	establishment of localization								
GO:0009152	P	purine ribonucleotide biosynthetic process								
GO:0051179	P	localization								
GO:0009260	P	ribonucleotide biosynthetic process								
GO:0006164	P	purine nucleotide biosynthetic process								
GO:0006812	P	cation transport								
GO:0009165	P	nucleotide biosynthetic process								
GO:0015991	P	ATP hydrolysis coupled proton transport								
GO:0015988	P	energy coupled proton transport, against electrochemical gradient								
GO:0006811	P	ion transport								
GO:0019684	P	photosynthesis, light reaction								
GO:0046034	P	ATP metabolic process								
GO:0055085	P	transmembrane transport								
GO:0009144	P	purine nucleoside triphosphate metabolic process								
GO:0009199	P	ribonucleoside triphosphate metabolic process								
GO:0009205	P	purine ribonucleoside triphosphate metabolic process								
GO:0009141	P	nucleoside triphosphate metabolic process								
GO:0009150	P	purine ribonucleotide metabolic process								
GO:0009259	P	ribonucleotide metabolic process								
GO:0006163	P	purine nucleotide metabolic process								
GO:0006508	P	proteolysis								
GO:0009117	P	nucleotide metabolic process								
GO:0006753	P	nucleoside phosphate metabolic process								
GO:0055086	P	nucleobase, nucleoside and nucleotide metabolic process								
GO:0055114	P	oxidation reduction								
GO:0046483	P	heterocycle metabolic process								
GO:0016310	P	phosphorylation								
GO:0003924	F	GTPase activity								
GO:0004252	F	serine-type endopeptidase activity								
GO:0015077	F	monovalent inorganic cation transmembrane transporter activity								
GO:0015078	F	hydrogen ion transmembrane transporter activity								
GO:0017171	F	serine hydrolase activity								
GO:0008236	F	serine-type peptidase activity								
GO:0022890	F	inorganic cation transmembrane transporter activity								
GO:0046933	F	hydrogen ion transporting ATP synthase activity, rotational mechanism								
GO:0046961	F	proton-transporting ATPase activity, rotational mechanism								
GO:0005525	F	GTP binding								
GO:0032561	F	guanyl ribonucleotide binding								
GO:0019001	F	guanyl nucleotide binding								
GO:0019829	F	cation-transporting ATPase activity								
GO:0008324	F	cation transmembrane transporter activity								
GO:0017111	F	nucleoside-triphosphatase activity								
GO:0042625	F	ATPase activity, coupled to transmembrane movement of ions								
GO:0016818	F	hydrolase activity, acting on acid anhydrides, in phosphorus-containing anhydrides								
GO:0016462	F	pyrophosphatase activity								
GO:0016787	F	hydrolase activity								
GO:0016817	F	hydrolase activity, acting on acid anhydrides								
GO:0004175	F	endopeptidase activity								
GO:0042626	F	ATPase activity, coupled to transmembrane movement of substances								
GO:0043492	F	ATPase activity, coupled to movement of substances								
GO:0015075	F	ion transmembrane transporter activity								
GO:0016820	F	substances								
GO:0022891	F	substrate-specific transmembrane transporter activity								
GO:0015405	F	P-P-bond-hydrolysis-driven transmembrane transporter activity								
GO:0015399	F	primary active transmembrane transporter activity								
GO:0046906	F	tetrapyrrole binding								
GO:0022892	F	substrate-specific transporter activity								
GO:0022857	F	transmembrane transporter activity								
GO:0009055	F	electron carrier activity								
GO:0070011	F	peptidase activity, acting on L-amino acid peptides								
GO:0008135	F	translation factor activity, nucleic acid binding								

GO Term	Aspect	Description	OT	ChR	SM	PP	PT	SB	OS	AT
GO:0022804	F	active transmembrane transporter activity								
GO:0008233	F	peptidase activity								
GO:0003746	F	translation elongation factor activity								
GO:0005215	F	transporter activity								
GO:0032555	F	purine ribonucleotide binding								
GO:0032553	F	ribonucleotide binding								
GO:0017076	F	purine nucleotide binding								
GO:0042623	F	ATPase activity, coupled								
GO:0016887	F	ATPase activity								
GO:0031976	C	plastid thylakoid								
GO:0055035	C	plastid thylakoid membrane								
GO:0009535	C	chloroplast thylakoid membrane								
GO:0009534	C	chloroplast thylakoid								
GO:0031984	C	organelle subcompartment								
GO:0044435	C	plastid part								
GO:0044434	C	chloroplast part								
GO:0042651	C	thylakoid membrane								
GO:0009536	C	plastid								
GO:0044436	C	thylakoid part								
GO:0009507	C	chloroplast								
GO:0034357	C	photosynthetic membrane								
GO:0009579	C	thylakoid								
GO:0031090	C	organelle membrane								
GO:0043234	C	protein complex								
GO:0044444	C	cytoplasmic part								
GO:0009521	C	photosystem								
GO:0043231	C	intracellular membrane-bounded organelle								
GO:0043227	C	membrane-bounded organelle								
GO:0044425	C	membrane part								
GO:0044422	C	organelle part								
GO:0044446	C	intracellular organelle part								
GO:0016469	C	proton-transporting two-sector ATPase complex								
GO:0005737	C	cytoplasm								
GO:0032991	C	macromolecular complex								
GO:0045259	C	proton-transporting ATP synthase complex								
GO:0016020	C	membrane								
GO:0043229	C	intracellular organelle								
GO:0043226	C	organelle								
GO:0005622	C	intracellular								
GO:0009523	C	photosystem II								
GO:0033178	C	proton-transporting two-sector ATPase complex, catalytic domain								
GO:0044424	C	intracellular part								
GO:0045261	C	proton-transporting ATP synthase complex, catalytic core F(1)								
GO:0016021	C	integral to membrane								
GO:0031224	C	intrinsic to membrane								
GO:0044464	C	cell part								
GO:0005623	C	cell								
GO:0009522	C	photosystem I								
GO:0009539	C	photosystem II reaction center								
GO:0034645	P	cellular macromolecule biosynthetic process								
GO:0009059	P	macromolecule biosynthetic process								
GO:0010467	P	gene expression								
GO:0044249	P	cellular biosynthetic process								
GO:0006412	P	translation								
GO:0009058	P	biosynthetic process								
GO:0043170	P	macromolecule metabolic process								
GO:0044238	P	primary metabolic process								
GO:0006350	P	transcription								
GO:0044260	P	cellular macromolecule metabolic process								
GO:0019538	P	protein metabolic process								
GO:0044237	P	cellular metabolic process								
GO:0006139	P	nucleobase, nucleoside, nucleotide and nucleic acid metabolic process								
GO:0044267	P	cellular protein metabolic process								
GO:0008152	P	metabolic process								
GO:0009987	P	cellular process								
GO:0006807	P	nitrogen compound metabolic process								
GO:0008547	F	protein-synthesizing GTPase activity								
GO:0034062	F	RNA polymerase activity								
GO:0003899	F	DNA-directed RNA polymerase activity								
GO:0003735	F	structural constituent of ribosome								
GO:0005198	F	structural molecule activity								
GO:0016779	F	nucleotidyltransferase activity								
GO:0005840	C	ribosome								
GO:0030529	C	ribonucleoprotein complex								
GO:0043232	C	intracellular non-membrane-bounded organelle								
GO:0043228	C	non-membrane-bounded organelle								
GO:0022904	P	respiratory electron transport chain								
GO:0042773	P	ATP synthesis coupled electron transport								
GO:0045333	P	cellular respiration								
GO:0015980	P	energy derivation by oxidation of organic compounds								
GO:0006120	P	mitochondrial electron transport, NADH to ubiquinone								

GO Term	Aspect	Description	OT	ChR	SM	PP	PT	SB	OS	AT
GO:0042775	P	mitochondrial ATP synthesis coupled electron transport								
GO:0006793	P	phosphorus metabolic process								
GO:0006796	P	phosphate metabolic process								
GO:0006118	P	electron transport								
GO:0003954	F	NADH dehydrogenase activity								
GO:0050136	F	NADH dehydrogenase (quinone) activity								
GO:0008137	F	NADH dehydrogenase (ubiquinone) activity								
GO:0016655	F	acceptor								
GO:0016651	F	oxidoreductase activity, acting on NADH or NADPH								
GO:0003902	F	DNA-directed RNA polymerase III activity								
GO:0003900	F	DNA-directed RNA polymerase I activity								
GO:0003901	F	DNA-directed RNA polymerase II activity								
GO:0008462	F	endopeptidase Clp activity								
GO:0003936	F	hydrogen-transporting two-sector ATPase activity								
GO:0033279	C	ribosomal subunit								
GO:0032774	P	RNA biosynthetic process								
GO:0006351	P	transcription, DNA-dependent								
GO:0016070	P	RNA metabolic process								
GO:0003677	F	DNA binding								
GO:0016772	F	transferase activity, transferring phosphorus-containing groups								
GO:0003676	F	nucleic acid binding								
GO:0003824	F	catalytic activity								
GO:0016740	F	transferase activity								
GO:0044281	P	small molecule metabolic process								
GO:0006510	P	ATP-dependent proteolysis								
GO:0042777	P	plasma membrane ATP synthesis coupled proton transport								
GO:0010255	P	glucose mediated signaling pathway								
GO:0009757	P	hexose mediated signaling								
GO:0000022	P	mitotic spindle elongation								
GO:0051231	P	spindle elongation								
GO:0007052	P	mitotic spindle organization								
GO:0009749	P	response to glucose stimulus								
GO:0006996	P	organelle organization								
GO:0007051	P	spindle organization								
GO:0034284	P	response to monosaccharide stimulus								
GO:0009746	P	response to hexose stimulus								
GO:0010182	P	sugar mediated signaling pathway								
GO:0030004	P	cellular monovalent inorganic cation homeostasis								
GO:0009756	P	carbohydrate mediated signaling								
GO:0006414	P	translational elongation								
GO:0000226	P	microtubule cytoskeleton organization								
GO:0055067	P	monovalent inorganic cation homeostasis								
GO:0009658	P	chloroplast organization								
GO:0016043	P	cellular component organization								
GO:0065003	P	macromolecular complex assembly								
GO:0030003	P	cellular cation homeostasis								
GO:0007017	P	microtubule-based process								
GO:0055080	P	cation homeostasis								
GO:0043933	P	macromolecular complex subunit organization								
GO:0031047	P	gene silencing by RNA								
GO:0070271	P	protein complex biogenesis								
GO:0006461	P	protein complex assembly								
GO:0009657	P	plastid organization								
GO:0006873	P	cellular ion homeostasis								
GO:0000279	P	M phase								
GO:0055082	P	cellular chemical homeostasis								
GO:0050801	P	ion homeostasis								
GO:0022607	P	cellular component assembly								
GO:0007010	P	cytoskeleton organization								
GO:0000278	P	mitotic cell cycle								
GO:0009743	P	response to carbohydrate stimulus								
GO:0022403	P	cell cycle phase								
GO:0016458	P	gene silencing								
GO:0034622	P	cellular macromolecular complex assembly								
GO:0048878	P	chemical homeostasis								
GO:0010608	P	posttranscriptional regulation of gene expression								
GO:0040029	P	regulation of gene expression, epigenetic								
GO:0009793	P	embryonic development ending in seed dormancy								
GO:0019725	P	cellular homeostasis								
GO:0034621	P	cellular macromolecular complex subunit organization								
GO:0022402	P	cell cycle process								
GO:0070887	P	cellular response to chemical stimulus								
GO:0048316	P	seed development								
GO:0010154	P	fruit development								
GO:0019843	F	rRNA binding								
GO:0008553	F	hydrogen-exporting ATPase activity, phosphorylative mechanism								
GO:0032549	F	ribonucleoside binding								
GO:0015662	F	mechanism								
GO:0048038	F	quinone binding								
GO:0003723	F	RNA binding								

GO Term	Aspect	Description	OT	ChR	SM	PP	PT	SB	OS	AT
GO:0046872	F	metal ion binding								
GO:0051536	F	iron-sulfur cluster binding								
GO:0051540	F	metal cluster binding								
GO:0043167	F	ion binding								
GO:0043169	F	cation binding								
GO:0009840	C	chloroplastic endopeptidase Clp complex								
GO:0009368	C	endopeptidase Clp complex								
GO:0009570	C	chloroplast stroma								
GO:0009532	C	plastid stroma								
GO:0033180	C	proton-transporting V-type ATPase, V1 domain								
GO:0000428	C	DNA-directed RNA polymerase complex								
GO:0055029	C	nuclear DNA-directed RNA polymerase complex								
GO:0015934	C	large ribosomal subunit								
GO:0030880	C	RNA polymerase complex								
GO:0033176	C	proton-transporting V-type ATPase complex								
GO:0000221	C	vacuolar proton-transporting V-type ATPase, V1 domain								
GO:0016471	C	vacuolar proton-transporting V-type ATPase complex								
GO:0022625	C	cytosolic large ribosomal subunit								
GO:0070013	C	intracellular organelle lumen								
GO:0043233	C	organelle lumen								
GO:0031974	C	membrane-enclosed lumen								
GO:0005739	C	mitochondrion								
GO:0044455	C	mitochondrial membrane part								
GO:0044429	C	mitochondrial part								
GO:0031980	C	mitochondrial lumen								
GO:0005759	C	mitochondrial matrix								
GO:0005774	C	vacuolar membrane								
GO:0022626	C	cytosolic ribosome								
GO:0044437	C	vacuolar part								
GO:0044451	C	nucleoplasm part								
GO:0031981	C	nuclear lumen								
GO:0005743	C	mitochondrial inner membrane								
GO:0044445	C	cytosolic part								
GO:0031966	C	mitochondrial membrane								
GO:0005654	C	nucleoplasm								
GO:0005773	C	vacuole								
GO:0005740	C	mitochondrial envelope								
GO:0019866	C	organelle inner membrane								
GO:0005730	C	nucleolus								
GO:0044428	C	nuclear part								
GO:0044427	C	chromosomal part								
GO:0071704	P	organic substance metabolic process								
GO:0015977	P	carbon fixation								
GO:0045156	F	electron transporter, transferring electrons within the cyclic electron transport pathway of								
GO:0016168	F	chlorophyll binding								
GO:0016984	F	ribulose-bisphosphate carboxylase activity								
GO:0016491	F	oxidoreductase activity								
GO:0051539	F	4 iron, 4 sulfur cluster binding								
GO:0016831	F	carboxy-lyase activity								
GO:0016830	F	carbon-carbon lyase activity								
GO:0015935	C	small ribosomal subunit								
GO:0030076	C	light-harvesting complex								
GO:0030077	C	plasma membrane light-harvesting complex								
GO:0042716	C	plasma membrane-derived chromatophore								
GO:0044433	C	cytoplasmic vesicle part								
GO:0031982	C	vesicle								
GO:0031410	C	cytoplasmic vesicle								
GO:0009573	C	chloroplast ribulose bisphosphate carboxylase complex								
GO:0048492	C	ribulose bisphosphate carboxylase complex								
GO:0005829	C	cytosol								
GO:0031975	C	envelope								
GO:0031967	C	organelle envelope								
