# Supplementary figures and images for: A Census of Nuclear Cyanobacterial Recruits in the Plant Kingdom
Source: PLoS One. 2015 Mar 20;10(3):e0120527. doi: 10.1371/journal.pone.0120527 (PMC4368824; doi:10.1371/journal.pone.0120527)

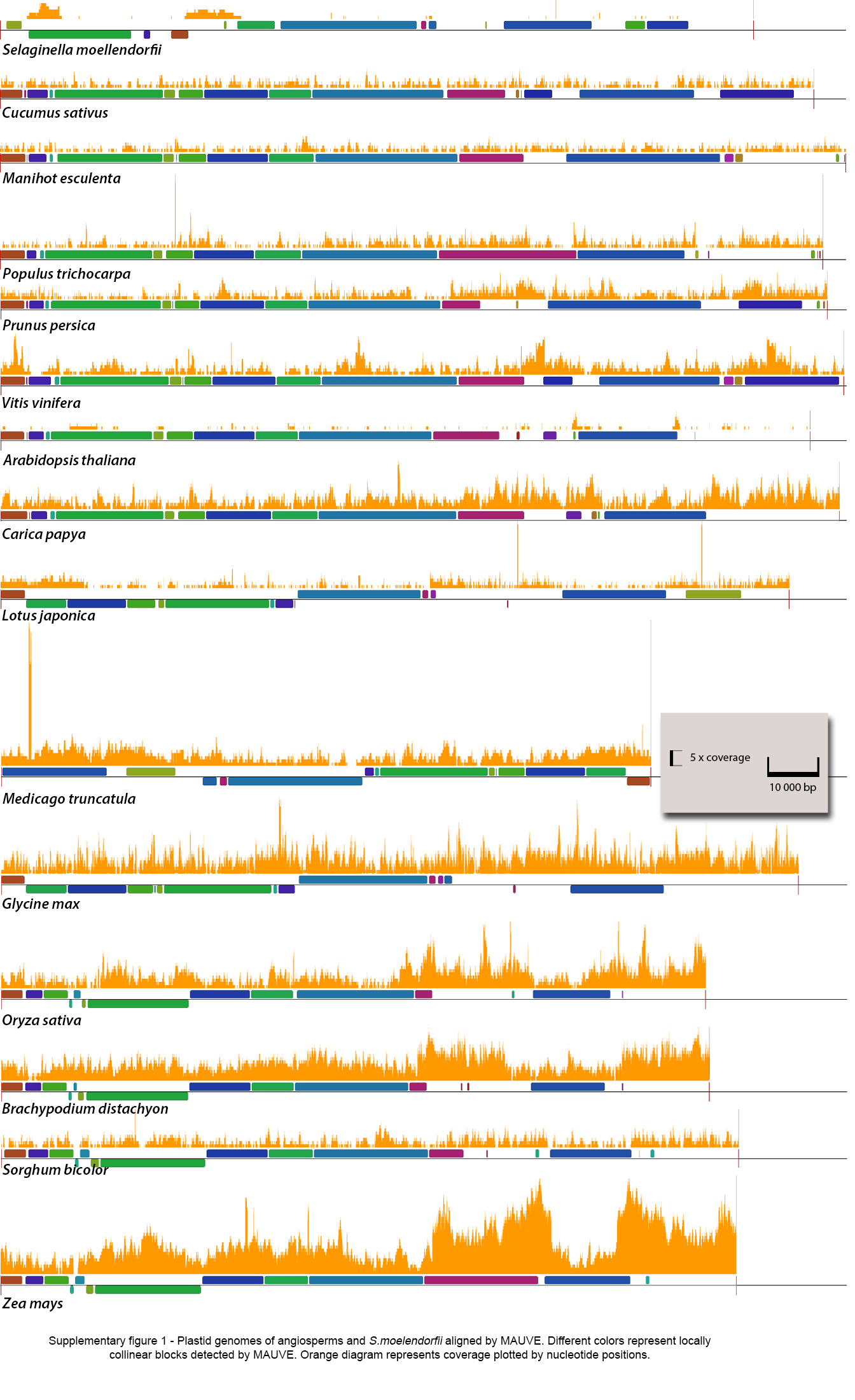

Supplement: S1 Fig — Different colors represent locally collinear blocks detected by MAUVE. Orange diagram represents coverage plotted by nucleotide positions. (TIF) [file pone.0120527.s003.tif]

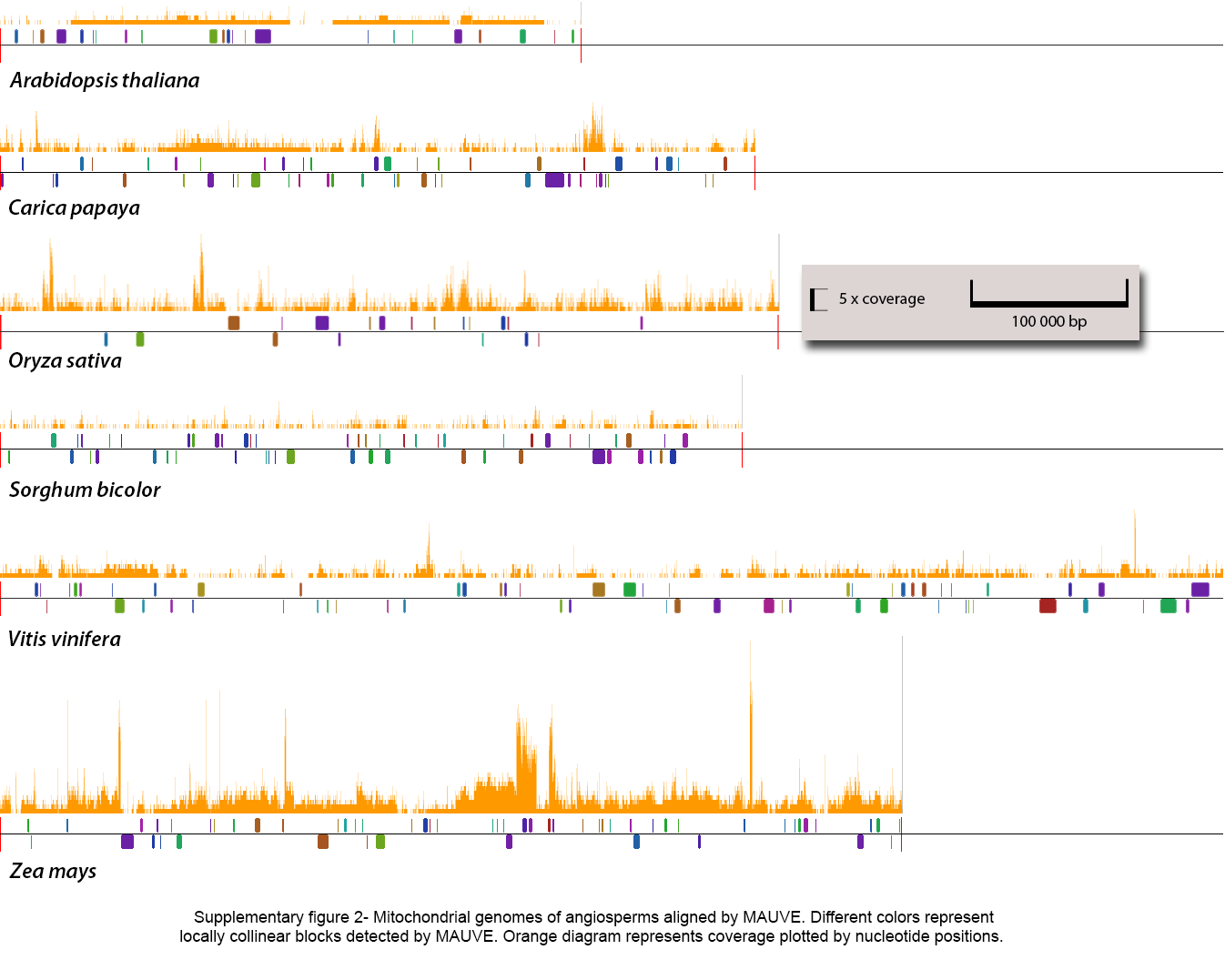

Supplement: S2 Fig — Different colors show locally collinear blocks (LCB as detected by MAUVE), oranges shows coverage as plotted against nucleotide positions. (TIF) [file pone.0120527.s004.tif]
